# Selection and Validation of qRT-PCR Internal Reference Genes to Study Flower Color Formation in *Camellia impressinervis*

**DOI:** 10.3390/ijms25053029

**Published:** 2024-03-06

**Authors:** Peilan Zhang, Shuying Chen, Siyu Chen, Yuanming Zhu, Yuqing Lin, Xinyu Xu, Zhongjian Liu, Shuangquan Zou

**Affiliations:** 1 College of Forestry, Fujian Agriculture and Forestry University, Fuzhou 350002, China; 2 Fujian Colleges and Universities Engineering Research Institute of Conservation and Utilization of Natural Bioresources, Fujian Agriculture and Forestry University, Fuzhou 350002, China; 3 Key Laboratory of National Forestry and Grassland Administration for Orchid Conservation and Utilization, College of Landscape Architecture and Art, Fujian Agriculture and Forestry University, Fuzhou 350002, China

**Keywords:** *Camellia impressinervis*, flower color, reference genes, qRT-PCR, *CiTUB* gene

## Abstract

Real-time quantitative PCR (qRT-PCR) is a pivotal technique for gene expression analysis. To ensure reliable and accurate results, the internal reference genes must exhibit stable expression across varied experimental conditions. Currently, no internal reference genes for *Camellia impressinervis* have been established. This study aimed to identify stable internal reference genes from eight candidates derived from different developmental stages of *C. impressinervis* flowers. We employed geNorm, NormFinder, and BestKeeper to evaluate the expression stability of these candidates, which was followed by a comprehensive stability analysis. The results indicated that *CiTUB*, a tubulin gene, exhibited the most stable expression among the eight reference gene candidates in the petals. Subsequently, *CiTUB* was utilized as an internal reference for the qRT-PCR analysis of six genes implicated in the petal pigment synthesis pathway of *C. impressinervis*. The qRT-PCR results were corroborated by transcriptome sequencing data, affirming the stability and suitability of *CiTUB* as a reference gene. This study marks the first identification of stable internal reference genes within the entire genome of *C. impressinervis*, establishing a foundation for future gene expression and functional studies. Identifying such stable reference genes is crucial for advancing molecular research on *C. impressinervis*.

## 1. Introduction

The *Camellia impressinervis*, classified within Sect. *Chrysantha* Chang of the Camellia genus, is predominantly endemic to southern China [1]. It is esteemed in traditional Chinese medicine for its diverse therapeutic properties, including anti-inflammatory, diuretic, anti-neoplastic, lipid-modulating, and glycemic regulatory effects [2,3]. Additionally, its distinct golden-yellow flowers confer ornamental value and render it a valuable genetic resource for breeding. The unique floral pigmentation is primarily attributed to the petal’s structural attributes and the composition and concentration of cellular pigments, notably flavonoids and carotenoids [4,5,6,7]. These pigments are synthesized through complex enzymatic pathways: flavonoid biosynthesis necessitates enzymes including phenylalanine ammonia-lyase (PAL), cinnamate 4-hydroxylase (C4H), chalcone synthase (CHS), chalcone isomerase (CHI), flavanone 3-hydroxylase (F3H), and dihydroflavonol 4-reductase (DFR) [8,9,10,11]; carotenoid biosynthesis involves enzymes such as phytoene desaturase (PDS), carotenoid isomerase (CRTISO), lycopene ε-cyclase (LCYE), lycopene β-cyclase (LCYB), β-carotene hydroxylase (BCH), and zeaxanthin epoxidase (ZEP) [12,13,14,15,16]. Current literature on the molecular mechanisms influencing golden camellia flower coloration is scant. Notably, Jiang’s study demonstrated that *CnDFR* expression enhances polyphenol biosynthesis, positively correlating with polyphenol concentration and inversely with yellow pigmentation [17]. Similarly, elevated *CnF3′H* expression was observed to increase the total polyphenol and specific flavonol content in transgenic tobacco flowers [18]. Therefore, elucidating gene expression related to flower coloration in *C. impressinervis* is imperative for understanding the molecular basis of these phenotypic traits.

Real-time quantitative PCR (qRT-PCR) is prominently utilized owing to its specificity, sensitivity, and reproducibility for the quantitative analysis of gene expression [19,20,21]. However, the reliability of qRT-PCR results can be influenced by factors such as RNA quality, primer specificity, and the efficiencies of reverse transcription and amplification [22,23]. Consequently, the incorporation of one or more suitable internal reference genes for normalization is critical in the qRT-PCR process [24]. Commonly in botanical research, housekeeping genes like 18S ribosomal RNA (18S rRNA), elongation factor (EF), polyubiquitin (UBQ), glyceraldehyde-3-phosphate dehydrogenase (GAPDH), actin (ACT), and tubulin (TUB) are employed as reference genes [25,26,27,28]. Ideally, these reference genes should exhibit stable expression across different tissues, developmental stages, and experimental conditions [29]. However, studies reveal that no gene exhibits absolute stability; the perceived constancy of housekeeping genes is contingent upon specific experimental variables [30,31,32]. The most suitable internal reference genes for *Iris. lactea* var. *Chinensis* under cadmium stress are *EIF-5A* and *UBC*, while the most suitable internal reference genes under abiotic stress conditions are *TIP41*, *CYP*, *PGK*, *GAP*, and *PP2Acs*, such as salt stress, drought stress, cold stress, etc. [33,34]. Among different varieties and tissues of *Zanthoxylum bungeanum*, *UBQ* and *UBA* expression are the most stable, while in different stages of fruit, *UBQ* and *TIF* expression are the most stable [35]. In studying the expression of genes related to flower color synthesis, the internal reference genes used in each species were different. The expression of *CHS*, *DFR*, and *ANS* in the anthocyanin synthesis pathway was analyzed using *GAPDH* as the internal reference in *Schima superba*, and the results were consistent with the trend of anthocyanin accumulation in leaves [36]. Yoshihara used *AQP* as the internal reference to analyze the gene expression levels in the flavonoid/anthocyanin synthesis pathway of *Iris hollandica* [37,38]. Thus, selecting stably expressed internal reference genes tailored to specific experimental contexts is pivotal in gene expression analysis.

In this study, eight candidate genes (*Ci18S*, *CiACT*, *CiEF1α*, *CiEIF3*, *CiGAPDH*, *CiTUA*, *CiTUB*, *CiUBQ*) were identified based on genomic and transcriptomic analyses of *C. impressinervis*. The tools such as geNorm, NormFinder, and BestKeeper were employed to assess their stability. The stability ranking of three software reference genes was comprehensively analyzed to screen out the most stable genes in the petals of *C. impressinervis*. The stability of the internal reference genes was verified by detecting the expression of key structural genes in the flavonoid and carotenoid metabolic pathways of *C. impressinervis*. The final identified the most stable internal reference genes suitable for the expression of genes related to *C. impressinervis* while also providing a reference and basis for the study of target gene expression.

## 2. Results

### 2.1. Petal Pigment Categories

**Figure 1:**
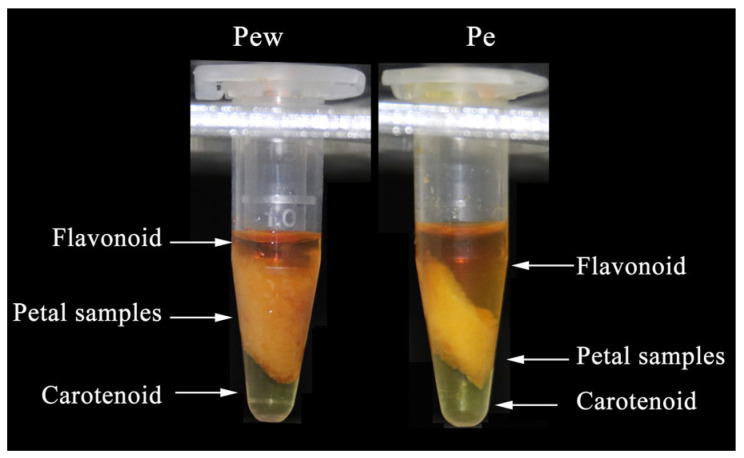
Identification of the petal pigment. Flavonoids are located in the upper layer, carotenoids are located in the lower layer, and the middle layer is the petal sample. Pew, outer petal; Pe, inner petal.

To elucidate the pigment composition of *C. impressinervis* petals, we analyzed the pigments from petals harvested at the blooming stage (Figure 1). The analysis revealed that both the outer and inner petals predominantly contain flavonoids and carotenoids. These pigments correspond closely with the observed phenotypic colors of the petals.

### 2.2. Petal Pigment Content

The content of flavonoids and carotenoids in the petals of *C. impressinervis* was quantified across various developmental stages (Table 1). The analysis indicated that the highest concentration of flavonoids in the outer petals was observed at the S1 stage (7.746 mg/g), while the peak for carotenoid content was at the S3 stage (0.112 mg/g). Similarly, in the inner petals, the maximum flavonoid content was at the S1 stage (7.845 mg/g), and the peak for carotenoids was at the S2 stage (0.149 mg/g). Notably, flavonoid concentrations at the S1 stage were significantly higher than those at S2 and S3 stages, demonstrating a decreasing trend with progression in developmental stages. Conversely, carotenoid content was significantly lower in the S1 stage compared to S2 and S3 stages, suggesting a trend of accumulation in later stages. This pattern indicates a predominant accumulation of flavonoids in the early stages of floral development and carotenoids in the later stages.

### 2.3. Primer Specificity Analysis

**Figure 2:**
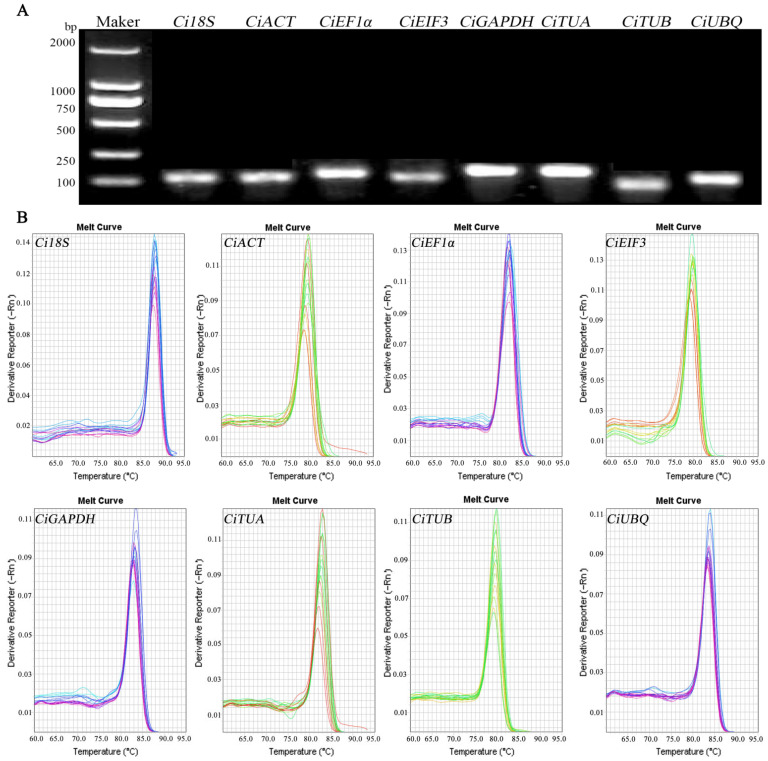
Performance of the amplification primers. (**A**) Amplification products obtained via qRT-PCR. (**B**) Melting curves of candidate genes.

Based on genome and transcriptome data, stable expression candidate reference genes were initially selected. The specificity of the candidate reference genes primers was evaluated to ensure the accuracy of qRT-PCR. Agarose gel electrophoresis revealed singular bands for all products, with no primer–dimer formations evident (Figure 2A), and melting curve analysis displayed single, distinct peaks for each primer pair (Figure 2B). These results confirm the high specificity of the primers, enabling them to uniquely amplify target internal reference gene products with good repeatability between amplification cycles. This validates their suitability for use in qRT-PCR.

The efficiency of primer amplification (*E*) was calculated based on the slope (*K*) and correlation coefficient (R^2^) derived from the standard curve. The efficiencies of the candidate genes ranged from 90.60% to 110.68% with all R2 values exceeding 0.98 (Table 2). These figures indicate robust primer amplification efficiency, satisfying the stringent criteria for qRT-PCR amplification efficiency.

### 2.4. Analysis of Candidate Gene Expression Abundance

**Figure 3:**
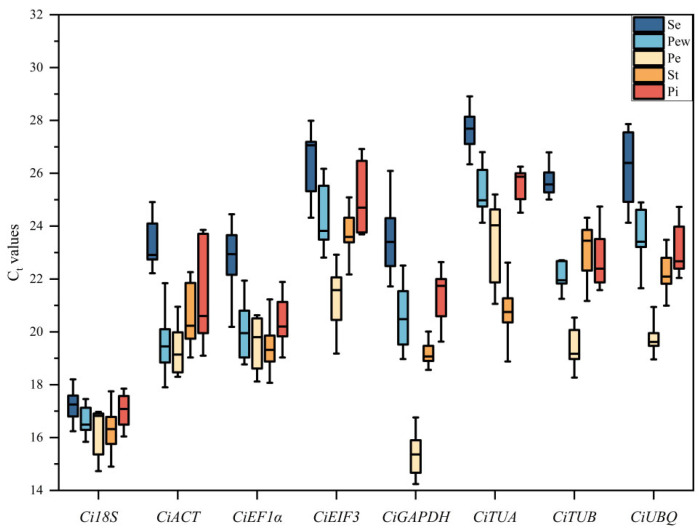
Distribution of Ct values for the candidate reference genes. Se, sepal; Pew, outer petal; Pe, inner petal; St, Stamen; Pi, pistil.

The threshold cycle (Ct) value is indicative of the gene expression levels in samples. In the floral organs of *C. impressinervis*, the Ct values of the candidate genes ranged from 14.242 to 28.914 (Figure 3). Within the inner petals, the lowest mean Ct value was observed for *CiGAPDH* (15.38), and the highest for *CiTUA* (23.05). Conversely, in the outer petals, pistils, and sepals, the lowest mean Ct value was recorded for *Ci18S* (16.66, 16.96, and 17.24, respectively), and the highest for *CiTUA* (25.00, 25.46, and 27.23, respectively). In the stamens, the lowest average Ct value was for *Ci18S* (16.33), while the highest was for *CiEIF3* (23.70). These results indicate a relatively high expression abundance of *Ci18S* and a low expression abundance of *CiTUA* across the floral tissues. Furthermore, the coefficient of variation for *Ci18S* was relatively low in the sepals, outer petals, and pistils, suggesting minimal fluctuation and preliminary evidence of its stability (Table 3). In contrast, *CiACT* exhibited the highest coefficient of variation in both outer petals and pistils, indicating a broad range of Ct value fluctuations and thus poor stability (Table 3).

### 2.5. geNorm Analysis

**Figure 4:**
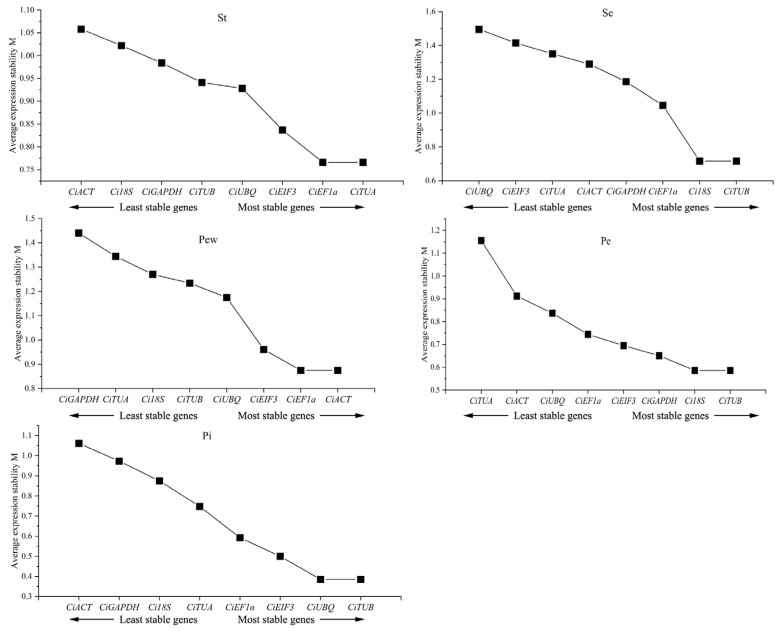
geNorm analysis of candidate reference genes’ expression stability. Stability increases progressively from left to right.

The geNorm analysis assesses the expression stability of candidate genes using the M value with values less than 1.5 reflecting high stability. The analysis revealed that all eight candidate genes across different floral parts registered M values below 1.5, indicating stable expression (Figure 4). Specifically, *Ci18S* and *CiTUB* showed the greatest stability in sepals and inner petals, while *CiACT* and *CiEF1α* were the most stable in outer petals. *CiEF1α* and *CiTUA* exhibited the highest stability in stamens and sepals, and *CiTUB* and *CiUBQ* exhibited the highest stability in pistils.

### 2.6. NormFinder Analysis

NormFinder evaluates candidate gene stability by calculating an expression stability value (SV), where a lower SV denotes greater stability. According to the analysis (Table 4), *CiTUB* consistently exhibited the lowest SV values and thus the highest stability in sepals, outer petals, inner petals, and pistils. Conversely, *CiTUA* showed the lowest SV and highest stability in stamens. These results suggest *CiTUB* as the most suitable reference gene for petals.

### 2.7. BestKeeper Analysis

BestKeeper analysis evaluates the expression stability of reference genes based on the standard deviation (SD) and coefficient of variation (CV) of the Ct values. Lower SD and CV values indicate better stability. According to the results (Table 5), the SD values of *CiTUB*, *CiGAPDH*, and *Ci18S* across different floral organs were all less than one, signifying their robust stability and their suitability as internal reference genes. Conversely, the SD values of *CiEIF3* in sepals, outer petals, inner petals, and pistils were all greater than one, indicating poor stability and rendering it unsuitable as an internal reference gene. Specifically, *CiTUB* demonstrated the smallest SD and highest stability among outer petals. In inner petals, *CiUBQ* showed the lowest SD, followed closely by *CiTUB*, suggesting *CiTUB* as a suitable reference gene in flower petals.

### 2.8. Comprehensive Analysis of Internal Parameter Stability

A comprehensive evaluation of the stability of eight candidate genes was conducted using geNorm, NormFinder, and BestKeeper analyses (Table 6). The results indicated that *CiTUB* consistently ranked highest in stability across sepals, outer petals, inner petals, and pistils, making it the most suitable internal reference gene for the study of *C. impressinervis*-related genes. *CiGAPDH* and *CiTUA* exhibited the least stability in outer and inner petals, respectively, and thus were deemed unsuitable as internal reference genes in this study.

### 2.9. Expression Analysis of Flower Color Related Genes

**Figure 5:**
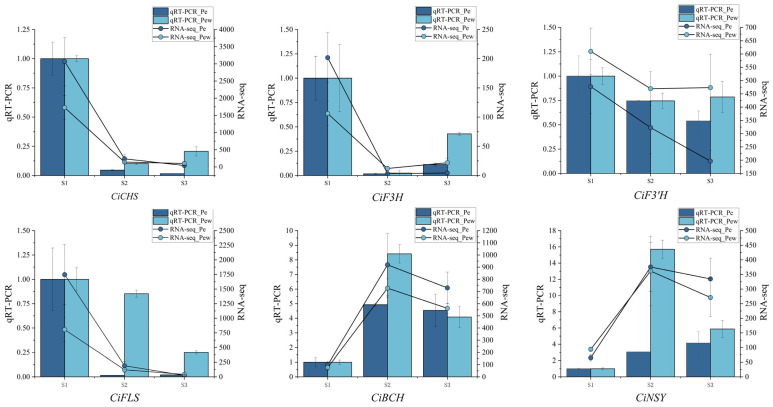
Expression analysis of genes involved in flower color formation. The bar chart represents qRT PCR data, and the line chart represents transcriptome data. The dark blue represents the inner petals, and light blue represents the outer petals.

This study utilized *CiTUB* as the internal reference gene to analyze the expression patterns of six genes implicated in the formation of flower color in *C. impressinervis*. There are four genes (*CiCHS*, *CiF3H*, *CiF3′H*, and *CiFLS*) associated with the flavonoid biosynthesis pathway and two genes (*CiBCH* and *CiNS*) associated with the carotenoid biosynthesis pathway. qRT-PCR analysis revealed that the relative expression levels of genes such as *CiCHS*, *CiF3H*, and *CiFLS* were highest during the S1 stage and generally exhibited a decreasing trend in later stages (Figure 5). The relative expression levels of *CiBCH* and *CiNSY* peaked in the S2 stage, showing an initial increase followed by a decrease. The expression trends of these six color-related genes in qRT-PCR analysis were consistent with those in transcriptome expression data, validating the reliability of *CiTUB* as a reference gene.

## 3. Discussion

Selecting appropriate reference genes is crucial for enhancing the reliability and accuracy of target gene expression analysis. The stability of internal reference genes varies across different plant parts and developmental stages [39,40]. To date, there have been no reports on the internal reference genes for *C. impressinervis*, which is commonly known as golden camellia. This study represents the first endeavor to screen and identify suitable internal reference genes for *C. impressinervis*, utilizing eight candidate genes derived from genomic and transcriptomic data. The analysis focused on assessing the stability of these reference genes across various parts and developmental stages of the plant.

In terms of expression abundance, *Ci18S* consistently exhibited a low average Ct value across all samples, coupled with minimal fluctuation, indicating its high expression level and robust stability. Notably, lower Ct values are indicative of higher gene expression levels. The 18S rRNA gene is known for its high expression across a wide array of plant tissues, including *C. impressinervis* [41,42], and it has been frequently employed as an internal reference gene in diverse plant species, such as *Narcissus pseudonarcissus* and *Solanum melongena* [41,43].

To further refine the selection of reference genes, three distinct algorithms—geNorm, NormFinder, and BestKeeper—were utilized to evaluate the stability of the candidate genes. Given the inherent differences in these algorithms, the recommended optimal reference genes varied for different plant parts [44]. Consequently, a comprehensive analysis was conducted to derive a stability ranking. This analysis concluded that *CiTUB* is the most suitable reference gene for sepals, petals, and pistils, while *CiTUA* is preferred for stamen.

Tubulin is closely related to intracellular material transport, maintaining cell shape, cell movement, mitosis, and other life activities [45]. Notably, *TUB* is consistently expressed across a variety of plant species and animals and has been utilized in gene expression analyses. In *Galeruca daurica*, *α-TUB* can serve as an internal reference gene in different tissues [46]. *GAPDH* and *TUB* are the optimal reference genes during the development of flower buds in Chinese cabbage (*Brassica rapa* L. ssp. *pekinensis*) [47]. *TUB* is also considered one of the most stable genes in *Platycladus orientalis* at all developmental stages and under all stress conditions [48]. The predominant pigments in plant petals include flavonoids, carotenoids, and betalains. Our investigation into the pigmentation of *C. impressinervis* petals confirmed the presence of flavonoids and carotenoids, which corresponded well with the observed petal color and phenotype. Quantitative analysis revealed a higher concentration of flavonoids compared to carotenoids. Notably, flavonoids predominantly accumulated during the initial S1 stage of development and subsequently diminished, while carotenoids peaked in the S2 stage, displaying an initial increase followed by a decrease. These dynamics underscore the complex regulation and temporal distribution of pigment synthesis in *C. impressinervis*. The synthesis of these pigments is regulated by various genes, necessitating the use of stable internal reference genes for accurate expression analysis. In the context of pigment-related gene expression, diverse internal reference genes have been employed across studies. For instance, researchers have used 18S rRNA for analyzing anthocyanin synthesis-related gene expression in *Dendrobium hybrids* [49], *ACT* for anthocyanin-related genes in *Muscari armeniacum* [50], and *TUB* for flavonoid and anthocyanin pathways in *Ipomoea batatas* [51]. In this study, we selected genes associated with flavonoid and carotenoid biosynthesis for expression analysis, utilizing *CiTUB* as the internal reference. The consistency between our qRT-PCR results and transcriptome sequencing data corroborates the stability of *CiTUB* as a reference gene. Furthermore, the concordance of expression trends in genes related to color formation and pigment content, such as *CiCHS*, *CiF3H*, *CiF3′H*, *CiFLS*, *CiBCH*, and *CiNSY*, highlights their significant roles in the development of *C. impressinervis*.

Homologous genes tend to be more conserved among closely related species. Therefore, *CiTUB*, chosen as the internal reference gene in this study, not only facilitates the expression analysis of anthocyanin synthesis-related genes within various *C. impressinervis* color varieties but also offers a reference for selecting internal reference genes for similar gene types in related species. Additionally, the stable expression of *CiTUB* across the sepals, petals, and pistils of *C. impressinervis* endorses its broader applicability as an internal reference gene for inter-organ target gene expression analysis within this species.

## 4. Materials and Methods

### 4.1. Plant Materials

**Figure 6:**
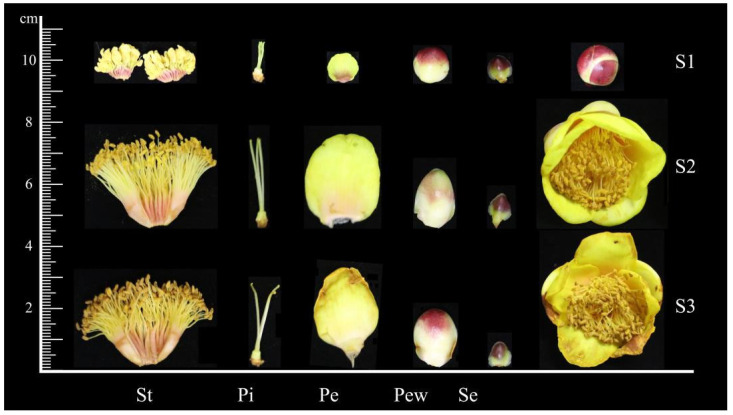
The development process of *C. impressinervis*. S1, young stage; S2, blooming stage; S3, decay stage; Se, sepal; Pew, outer petal; Pe, inner petal; St, Stamen; Pi, pistil.

*C. impressinervis* was selected as the experimental material and cultivated at Lingtou Oil Tea Farm, Jin’an District, Fuzhou City, Fujian Province, China, since 2014. Samples were collected in March 2022. Based on the flowering stages of *C. impressinervis* (Figure 6), the developmental cycle was categorized into three distinct periods: young stage (S1), blooming stage (S2), and decay stage (S3). The collected samples were segregated into five parts: outer petal (Pew), inner petal (Pe), sepal (Se), pistil (Pi), and stamen (St). Post-collection, samples were immediately placed in 5 mL sterile, enzyme-free centrifuge tubes and snap-frozen in liquid nitrogen. Three biological replicates were taken from each sample and stored at −80 °C for further analysis.

### 4.2. Identification of the Petal Pigment Categories

The pigmentation of the outer and inner petals was identified following Yuan’s method [52]. Fresh petals (25 mg) from the blooming period were ground into powder in liquid nitrogen, which was followed by the addition and mixing of 200 μL anhydrous methanol for 10 min. Subsequently, 200 μL water and 200 μL dichloromethane were added, thoroughly mixed, and allowed to settle for five minutes. The mixture was then centrifuged at 12,000 rpm for three minutes.

### 4.3. Determination of Flavonoids and Carotenoids Content

Visible spectrophotometry was employed to determine the flavonoids and carotenoids content in the inner and outer petals of *C. impressinervis* across different stages. Flavonoids and carotenoids were extracted using kits: UPLC-MS-4291 (Plant Flavonoids Content Assay Kit) and UPLC-MS-4268 (Plant Carotenoids Content Assay Kit). The absorbance at 470 nm and 440 nm was measured using a spectrophotometer to calculate the content.

### 4.4. RNA Extraction and cDNA Synthesis

Total RNA was extracted from *C. impressinervis* using the TIANGEN Extraction Kit (DP441). RNA integrity was verified through 1% agarose gel electrophoresis, and RNA concentration and purity were assessed using a NanoDrop 2000 microspectrophotometer (Thermo Fisher Scientific, Shanghai, China). Reverse transcription was conducted using the Hifair^®^ III 1st Strand cDNA Synthesis Kit (gDNA digest plus), employing a 20 μL reaction volume. The reaction conditions were as follows: 5 min at 25 °C, 15 min at 55 °C, and 5 min at 85 °C. The resulting cDNA was stored at −20 °C for downstream applications.

### 4.5. Primer Design for Candidate Genes and Flower Color Related Genes

Eight candidate genes (*Ci18S*, *CiACT*, *CiEF1α*, *CiGAPDH*, *CiEIF3*, *CiTUA*, *CiTUB*, *CiUBQ*) were screened from the genomic (PRJCA020809, https://ngdc.cncb.ac.cn/, accessed on 15 November 2023) and transcriptomic (PRJCA022871, accessed on 1 February 2024) data of *C. impressinervis*. Additionally, six genes related to floral pigment biosynthesis pathways (*CiCHS*, *CiFLS*, *CiF3′H*, *CiF3H*, *CiBCH*, *CiNSY*) were selected based on transcriptome sequencing KEGG data (PRJCA022871). Primers for qRT-PCR were designed using Primer Premier 5 [32] and synthesized by Sangon Biotech (Shanghai, China) Co., Ltd. The sequences of these primers are provided in Table 7.

### 4.6. Candidate Reference Gene qRT-PCR Analysis

A mixed cDNA template from sepals, outer petals, inner petals, stamens, and pistils at different developmental stages was prepared in five concentration gradients (5^0^, 5^−1^, 5^−2^, 5^−3^, and 5^−4^ times the stock solution). To measure the Ct values for each candidate gene at various gradients were obtained a Hifair^®^ qPCR SYBR Green Master Mix (Low Rox Plus) kit, which was used for qRT-PCR on the Applied Biosystems 7500 Real-Time System (Thermo Fisher Scientific, Shanghai, China). The program settings are set according to the reagent instruction manual. The reaction mixture was prepared on ice, with three replicates for each concentration. Using the Ct values, the standard curve, slope (*K*), linear correlation coefficient (R^2^), and amplification efficiency (*E*) were calculated [53].

### 4.7. Stability Evaluation of Candidate Genes

The expression stability of candidate genes (*Ci18S*, *CiACT*, *CiEF1α*, *CiGAPDH*, *CiEIF3*, *CiTUA*, *CiTUB*, *CiUBQ*) in the sepals, outer petals, inner petals, stamens, and pistils of *C. impressinervis* at different developmental stages was assessed using three software programs: geNorm (an Excel add-in, MS Office version 2003), NormFinder (an Excel add-in, MS Office version 2003), and BestKeeper (an Excel add-in, MS Office version 2003). The geNorm and NormFinder analyses employed the 2^−∆∆CT^ method for calculations, whereas BestKeeper utilized Ct values for its analysis [54]. A comprehensive stability ranking of the candidate reference genes was subsequently determined based on the results from these three programs. The geometric mean across all rankings was used to calculate an overall expression stability ranking, facilitating the selection of the most suitable reference genes for different floral organs.

### 4.8. Verification of Stability of Candidate Internal Reference Genes

To verify the most suitable internal reference genes, the expression patterns of six genes related to floral pigment synthesis in *C. impressinervis* petals were analyzed using qRT-PCR. The relative expression levels of these genes at various developmental stages were calculated using the 2^−∆∆CT^ method. This analysis aimed to confirm the stability and appropriateness of the selected internal reference genes for accurate expression analysis.

### 4.9. Data Processing

Data were compiled and processed using Microsoft Excel 2022. Statistical analyses, including Duncan’s multiple range test, were conducted using IBM SPSS Statistics 25.0. Graphical representations of the data were generated using Origin 2021 software. These tools facilitated the thorough analysis and visualization of experimental results.

## 5. Conclusions

Leveraging extensive genomic and transcriptomic data from *C. impressinervis*, a rigorous screening process was employed to select candidate internal reference genes for qRT-PCR. The stability of these eight candidates at different developmental stages and within various floral organs was meticulously analyzed through qRT-PCR assays using geNorm, NormFinder, and BestKeeper. A comprehensive analysis revealed that the optimal reference genes varied among the different floral organs. Specifically, *CiTUB* emerged as the most suitable reference gene for sepals, petals, and pistils, while *CiTUA* was the most apt for stamens. This study marks the inaugural identification of stable internal reference genes throughout the entire genome of *C. impressinervis*, establishing a vital foundation for enhanced future research into gene expression and functionality within this species.

## Figures and Tables

**Table 1:** Analysis of petal pigment content at different developmental periods. Different lowercase letters in the same column indicate significant differences between samples (*p* < 0.05). S1, young stage; S2, blooming stage; S3, decay stage.

Sample	Flavonoids Content (mg/g)	Carotenoids Content (mg/g)
S1_Pew	7.746 ± 0.3217 a	0.030 ± 0.0022 d
S2_Pew	5.101 ± 0.6136 b	0.110 ± 0.0069 c
S3_Pew	3.592 ± 0.2200 c	0.112 ± 0.0060 c
S1_Pe	7.845 ± 0.3986 a	0.035 ± 0.0029 d
S2_Pe	5.661 ± 0.3800 b	0.149 ± 0.0043 a
S3_Pe	3.045 ± 0.5477 c	0.136 ± 0.0119 b

**Table 2:** Amplification parameters for candidate reference genes. *K*: slope; *E*: primer amplification efficiency; R^2^: correlation coefficient.

Gene	*K*	*E* (%)	R^2^	Product Length (bp)
*Ci18S*	−3.50	93.07	0.9979	102
*CiACT*	−3.14	108.2	0.9924	93
*CiEF1α*	−3.57	90.60	0.9978	119
*CiEIF3*	−3.51	92.71	0.9879	92
*CiGAPDH*	−3.55	91.29	0.9925	140
*CiTUA*	−3.09	110.68	0.9952	146
*CiTUB*	−3.43	95.68	0.9924	81
*CiUBQ*	−3.22	104.44	0.9911	117

**Table 3:** The Ct value analysis of the candidate reference genes. SD: standard deviation; CV: coefficient of variation.

Sample		*Ci18S*	*CiACT*	*CiEF1α*	*CiEIF3*	*CiGAPDH*	*CiTUA*	*CiTUB*	*CiUBQ*
Se	average value	17.24	23.3	22.8	26.39	23.51	27.23	25.73	26.26
	SD	0.65	0.94	1.33	1.3	1.33	1.05	0.56	1.43
	CV (%)	3.76	4.04	5.84	4.92	5.64	3.87	2.17	5.44
Pew	average value	16.66	19.71	20.05	24.35	20.59	25	22.03	23.57
	SD	0.54	1.37	1.08	1.23	1.23	1.54	0.56	1.04
	CV (%)	3.25	6.96	5.4	5.06	5.95	6.14	2.55	4.43
Pe	average value	16.22	19.3	19.25	21.3	15.38	23.05	19.35	19.78
	SD	0.91	1.01	1.43	1.28	0.85	1.79	0.78	0.7
	CV (%)	5.6	5.24	7.44	6.03	5.53	7.76	4.04	3.53
St	average value	16.33	20.61	19.39	23.7	19.24	21.09	23	22.27
	SD	0.86	1.28	0.95	0.96	0.49	1.56	1.13	0.76
	CV (%)	5.29	6.19	4.88	4.05	2.53	7.41	4.91	3.43
Pi	average value	16.96	21.28	20.37	25.04	21.3	25.46	22.74	23.11
	SD	0.68	1.95	0.96	1.38	1.1	0.7	1.09	1.07
	CV (%)	3.98	9.16	4.73	5.52	5.16	2.75	4.79	4.62

**Table 4:** Candidate reference genes in NormFinder. SV: stable value.

Rank	Se		Pew		Pe		St		Pi	
	Gene	SV	Gene	SV	Gene	SV	Gene	SV	Gene	SV
1	*CiTUB*	0.16	*CiTUB*	0.23	*CiTUB*	0.12	*CiTUA*	0.16	*CiTUB*	0.11
2	*Ci18S*	0.25	*Ci18S*	0.26	*CiEIF3*	0.19	*CiEIF3*	0.19	*CiEF1α*	0.12
3	*CiGAPDH*	0.25	*CiEF1α*	0.27	*Ci18S*	0.21	*CiEF1α*	0.22	*CiEIF3*	0.14
4	*CiEF1α*	0.30	*CiEIF3*	0.29	*CiACT*	0.22	*CiUBQ*	0.25	*CiUBQ*	0.15
5	*CiACT*	0.30	*CiUBQ*	0.34	*CiGAPDH*	0.24	*CiGAPDH*	0.26	*CiACT*	0.23
6	*CiTUA*	0.34	*CiACT*	0.34	*CiUBQ*	0.26	*CiTUB*	0.26	*CiTUA*	0.28
7	*CiEIF3*	0.36	*CiTUA*	0.35	*CiEF1α*	0.34	*CiACT*	0.27	*CiGAPDH*	0.36
8	*CiUBQ*	0.37	*CiGAPDH*	0.36	*CiTUA*	0.43	*Ci18S*	0.27	*Ci18S*	0.37

**Table 5:** Candidate reference genes in BestKeeper. Min: minimum value; Max: maximum value; SD: standard deviation; CV: coefficient of variation.

Sample	Rank	Gene	Min	Max	SD (±Cq)	CV (%Cq)
Pe	1	*CiUBQ*	18.96	20.94	0.53	2.68
	2	*CiTUB*	18.27	20.54	0.63	3.25
	3	*CiGAPDH*	14.24	16.76	0.66	4.29
	4	*Ci18S*	14.73	16.97	0.77	4.75
	5	*CiEF1α*	18.12	20.63	0.80	4.08
	6	*CiACT*	18.30	20.95	0.82	4.25
	7	*CiEIF3*	19.18	22.92	1.01	4.75
	8	*CiTUA*	21.06	25.20	1.30	5.57
Pew	1	*CiTUB*	21.25	22.71	0.44	1.99
	2	*Ci18S*	15.84	17.46	0.47	2.80
	3	*CiUBQ*	21.65	24.90	0.79	3.34
	4	*CiTUA*	24.13	26.80	0.83	3.26
	5	*CiEF1α*	18.77	21.94	0.88	4.38
	6	*CiGAPDH*	18.97	22.51	0.98	4.76
	7	*CiEIF3*	22.81	26.17	1.03	4.21
	8	*CiACT*	17.90	21.84	1.03	5.24
St	1	*CiGAPDH*	18.56	20.01	0.39	2.04
	2	*CiUBQ*	20.99	23.48	0.63	2.81
	3	*Ci18S*	14.90	17.75	0.68	4.17
	4	*CiTUA*	18.88	22.62	0.70	3.35
	5	*CiEF1α*	18.07	21.23	0.70	3.63
	6	*CiEIF3*	22.17	25.09	0.73	3.08
	7	*CiTUB*	21.17	24.32	0.94	4.11
	8	*CiACT*	19.03	22.26	1.12	5.41
Pi	1	*CiTUA*	24.51	26.25	0.56	2.19
	2	*Ci18S*	16.04	17.85	0.57	3.34
	3	*CiEF1α*	19.03	21.89	0.81	3.96
	4	*CiTUB*	21.58	24.74	0.89	3.90
	5	*CiUBQ*	22.04	24.73	0.91	3.95
	6	*CiGAPDH*	19.63	22.64	0.93	4.36
	7	*CiEIF3*	23.69	26.92	1.20	4.78
	8	*CiACT*	19.10	23.86	1.66	7.81
Se	1	*CiTUB*	25.01	26.79	0.44	1.71
	2	*Ci18S*	16.24	18.20	0.52	3.03
	3	*CiTUA*	26.34	28.91	0.68	2.47
	4	*CiACT*	22.22	24.91	0.78	3.36
	5	*CiGAPDH*	21.72	26.09	0.99	4.23
	6	*CiEF1α*	20.19	24.45	1.03	4.53
	7	*CiEIF3*	24.32	27.99	1.09	4.14
	8	*CiUBQ*	24.13	27.86	1.24	4.73

**Table 6:** Stability of the comprehensive analysis of candidate reference genes.

Rank	Se		Pew		Pe		St		Pi	
	Gene	Geomean of Ranking Values	Gene	Geomean of Ranking Values	Gene	Geomean of Ranking Values	Gene	Geomean of Ranking Values	Gene	Geomean of Ranking Values
1	*CiTUB*	1.00	*CiTUB*	1.71	*CiTUB*	1.26	*CiTUA*	1.59	*CiTUB*	1.59
2	*Ci18S*	1.59	*CiEF1a*	2.47	*Ci18S*	2.29	*CiEF1a*	2.47	*CiUBQ*	2.71
3	*CiGAPDH*	3.91	*Ci18S*	2.88	*CiUBQ*	3.30	*CiGAPDH*	3.11	*CiEF1α*	2.88
4	*CiEF1a*	4.16	*CiACT*	3.63	*CiGAPDH*	3.56	*CiUBQ*	3.17	*CiTUA*	3.11
5	*CiACT*	4.64	*CiUBQ*	3.91	*CiEIF3*	3.83	*CiEIF3*	3.30	*CiEIF3*	3.98
6	*CiTUA*	4.76	*CiEIF3*	4.38	*CiACT*	5.52	*Ci18S*	5.52	*Ci18S*	4.58
7	*CiEIF3*	7.00	*CiTUA*	5.81	*CiEF1a*	5.59	*CiTUB*	5.94	*CiGAPDH*	6.65
8	*CiUBQ*	8.00	*CiGAPDH*	7.27	*CiTUA*	8.00	*CiACT*	7.65	*CiACT*	6.84

**Table 7:** Primer sequences of color-related genes for qRT-PCR. F: forward; R: reverse.

Gene	Gene ID	Primer Sequences (5′–3′)	Purpose
*Ci18S*	*JinHuaCha00367404*	*F: CGTTCGTCTGGCTTCTTAGTCCTTC*	Reference gene
		*R: AACTCGCACAAACCAAACACAACTC*	
*CiACT*	*JinHuaCha00305809*	*F: CTCTCGTCTTCTCCGTCTCCTCAC*	
		*R: AGCCTTCACCATTCCAGTTCCATTG*	
*CiEF1α*	*JinHuaCha00372976*	*F: TCGATTGCCACACTTCCCACATTG*	
		*R: CCCAGCGTCACCGTTCTTCAAG*	
*CiEIF3*	*JinHuaCha00379840*	*F: ACCGGCTTATGCGTTATGCTCATC*	
		*R: TGGTTCATGGCTGCTGTATGTCAC*	
*CiGAPDH*	*JinHuaCha00022420*	*F: AGCAAGGACTGGAGAGGTGGAAG*	
		*R: TCAACAGTGGGAACACGGAAAGC*	
*CiTUA*	*JinHuaCha00361094*	*F: GACTGTTGGAGGAGGTGATGATGC*	
		*R: GGTGGAAGAGTTGGCGGTATGTTC*	
*CiTUB*	*JinHuaCha00329993*	*F: AGTTGAGAACGCCGATGAGTGTATG*	
		*R: GTGGTGAGCTTGAGTGTACGGAAG*	
*CiUBQ*	*JinHuaCha00084829*	*F: TGCAGAAGGACCCTCCCACATC*	
		*R: CCAGAAATACGCCTCCAGCATACG*	
*CiCHS*	*JinHuaCha00343068*	*F: TCCCAGATAGTGACGGTGCCATC*	Flower pigment synthesis-related genes
		*R: GTTCCAATCAGAGATGCCCAAGGG*	
*CiFLS*	*JinHuaCha00037405*	*F: ACCAGCAATCACCACCGTCAAAG*	
		*R: CAGCCTCCTCCACCATCCTCAC*	
*CiF3′H*	*JinHuaCha00381658*	*F: ATCTGCTCCGTCCATCTCTTCTCC*	
		*R: CTAGGTTCACTGCTGCCGCTTG*	
*CiF3H*	*JinHuaCha00051595*	*F: TGGAGGTGTTGTCTGAGGCTATGG*	
		*R: GAGGTCGGGTTGTGGGCATTTC*	
*CiBCH*	*JinHuaCha00348661*	*F: ACGAAGAGGAGGGTGAGCAAGAG*	
		*R: CTAGACATGACTGCGGCGACAAG*	
*CiNSY*	*JinHuaCha00334476*	*F: CGGTCCTGGCTGATGTCATTGC*	
		*R: CCACAACCCATTCCACACCCTATG*	

## Data Availability

The data presented in this study are available in the article.
